# Skin eruption involving bilateral breasts following radiation therapy for invasive ductal carcinoma of the left breast

**DOI:** 10.1097/JW9.0000000000000016

**Published:** 2022-04-06

**Authors:** Annika Belzer, Jennifer M. McNiff, Jonathan S. Leventhal

**Affiliations:** a Yale School of Medicine, New Haven, Connecticut; b Department of Dermatology, Yale School of Medicine, New Haven, Connecticut; c Department of Pathology, Yale School of Medicine, New Haven, Connecticut

**Keywords:** Koebnerization, lichen sclerosus, morphea, radiation therapy, radiation-induced morphea

## Case summary

A 64-year-old female with a diagnosis of invasive ductal carcinoma of the left breast was referred to Oncodermatology 6 months after completing radiation therapy (RT) for new onset eruption of bilateral breasts. Past medical history was notable for biopsy-proven extragenital and genital lichen sclerosus (LS). During RT, she was treated prophylactically with mometasone cream and developed only mild radiation-associated erythema with no new dermatologic findings for several months.

**Fig. 1.**
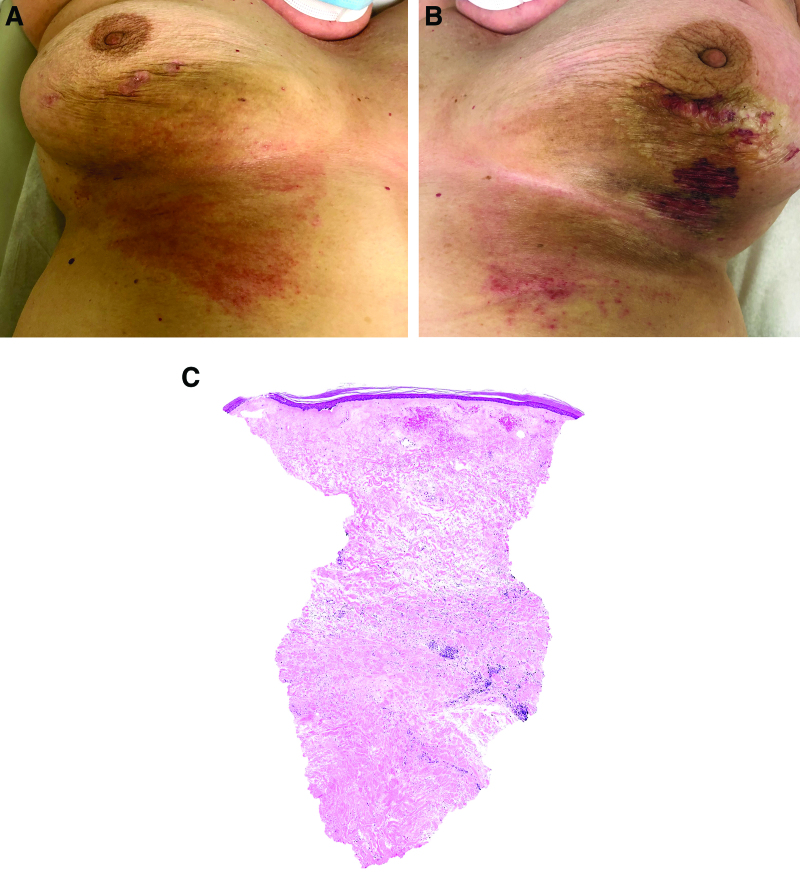
A 64-year-old woman with biopsy-proven lichen sclerosus presented to Oncodermatology for tender plaques of bilateral breasts several months after completing radiation therapy for invasive ductal carcinoma of the left breast. (A and B) Physical examination at initial presentation showed erythematous and hyperpigmented indurated, sclerotic plaques of the bilateral breasts, with ivory white papules and plaques and areas of purpura on the left greater than right. (C) Pathology showing flattened epidermis, homogenization of the papillary epidermis, and sclerosis of the deep reticular dermis.

The patient described initial erythema of the left breast that evolved into slightly tender plaques of bilateral breasts. Examination at the time of presentation revealed hyperpigmented indurated sclerotic plaques with a lilac rim of bilateral inframammary and lateral breasts, admixed with ivory white papules and plaques with purpura most pronounced on the irradiated left breast (Fig. 1A and B). Sclerotic plaques extended beyond the radiation field to the upper abdomen. Volume change of the breasts was not observed. Pathology demonstrated flattened epidermis with hyperkeratosis and homogenization of the papillary dermis (Fig. 1C). The deep reticular dermis revealed sclerosis with interstitial lymphocytes and plasma cells (Fig. 1C).

## What is your diagnosis?

1. Chronic radiation dermatitis
2. Inflammatory breast cancer
3. Cellulitis
4. Carcinoma en cuirasse
5. Radiation-induced morphea (RIM)/LS overlap

## Discussion

This case illustrates the scope of dermatologic adverse events (dAEs) following RT. Along with morphea and LS, inflammatory dermatoses including but not limited to graft versus host disease, erythema multiforme, Stevens-Johnson syndrome, and bullous pemphigoid can be triggered or exacerbated by RT. Further, RT can elicit koebnerization of papulosquamous conditions such as psoriasis and lichen planus.^1^

RIM is most common in women, with the majority of cases occurring within 1 year of receiving RT for treatment of breast cancer.^2^ Between 90–95% of patients undergoing RT will develop a cutaneous reaction and, due to a broad differential including radiation dermatitis, radiation recall dermatitis, cellulitis, inflammatory breast cancer, and cutaneous metastases (carcinoma en cuirasse), diagnosis of RIM is often delayed, resulting in preventable morbidity.^2^ Diagnosis can be supported clinically by the potential of RIM to extend beyond the irradiated field, as demonstrated in this case, in contrast to radiation-induced fibrosis, which remains localized to the irradiated field.^2^ Skin biopsy for dermatopathology confirms the diagnosis of RIM, preventing unnecessary imaging or treatment. Histologic findings during the “inflammatory phase” include perivascular and interstitial infiltration of lymphocytes, while the sclerotic “burnt out phase” is characterized by transition to extensive fibrosis with thickening of collagen fibers in the reticular dermis.^2^

LS is another chronic inflammatory dermatosis with similarities to morphea, including development of indurated, sclerotic plaques with lymphocytic infiltrate and fibrosis of the dermis on histology.^3^ Subtypes of LS include genital and extragenital, with extragenital being far less common.^4^ Prior data have shown an association of genital and extragenital LS with morphea.^4^ In a retrospective analysis of 472 patients diagnosed with morphea, 5.7% had comorbid LS. Of morphea cases with comorbid LS, the majority were extragenital (70.4%).^4^

The association of both morphea and LS with RT has been previously recognized, as has the increased prevalence of LS in patients with idiopathic morphea. However, there have been only a handful of cases of RIM-LS overlap. In a case series of RIM, Walsh et al.^2^ described 2 cases of RIM-LS overlap. Due to overlap in clinical presentation of these inflammatory dermatoses, histology can be used to distinguish the two; LS shows edema and hyalinization of the papillary dermis with underlying lymphocytic infiltrate and epidermal thinning, as opposed to histologic findings of the deeper reticular dermis in morphea.^2^ Although LS and morphea are both autoimmune inflammatory dermatoses that can develop in response to radiation, it is unclear whether the two are separate disease states or result from a common pathophysiologic phenomenon. We are hopeful that this case will stimulate further research into the subject.

This patient was treated with topical corticosteroids and methotrexate (off-label use) with clinical improvement, underscoring the importance of multidisciplinary care for patients who develop dAE following oncologic therapy. Timely dermatologic involvement may assist in prompt diagnosis and effective management of potentially treatment-limiting dAEs.

## Questions

Which of the following statements regarding RIM is true?

a. RIM typically develops during RT
b. RIM is dependent on radiation dose
c. RIM may extend beyond the radiation field
d. RIM is a precursor to angiosarcoma
e. RIM typically demonstrates atypical fibroblasts and vascular ectasia on pathology

Which of the following diagnostic tests is recommended when evaluating for potential RIM in a woman with a history of breast cancer?

a. Ultrasound of the breast
b. Punch biopsy of affected skin
c. Serum anti-nuclear antibody
d.Serum anti-scleroderma-70 (topoisomerase 1) antibody
e. No diagnostic tests indicated

## Author contributions

- AB: Conceptualization, Data Curation, Writing - Original Draft, Visualization.
- JMM: Data Curation, Writing - Review & Editing.
- JSL: Conceptualization, Writing - Review & Editing, Supervision.

## Conflicts of interest

The authors made the following disclosures: J.S.L.: serves on the advisory boards of La Roche-Posay and Sanofi and Regeneron Pharmaceuticals. A.B. and J.M.M.: None.

## Funding

None.

## Study approval

N/A.
