# Skin eruption involving bilateral breasts following radiation therapy for invasive ductal carcinoma of the left breast: Erratum

**DOI:** 10.1097/JW9.0000000000000052

**Published:** 2022-09-12

In the article entitled “Skin eruption involving bilateral breasts following radiation therapy for invasive ductal carcinoma of the left breast” (International Journal of Women’s Dermatology, June 2022, Volume 8, Issue 2), by Belzer et al., the correct answers to the questions were missing and have been provided below.

## What is your diagnosis?

1. Chronic radiation dermatitis
2. Inflammatory breast cancer
3. Cellulitis
4. Carcinoma en cuirasse
5. Radiation-induced morphea (RIM)/LS overlap

**Correct answer:** 5. Radiation-induced morphea (RIM)/LS overlap.

## Which of the following statements regarding RIM is true?

A. RIM typically develops during RT
B. RIM is dependent on radiation dose
C. RIM may extend beyond the radiation field
D. RIM is a precursor to angiosarcoma
E. RIM typically demonstrates atypical fibroblasts and vascular
ectasia on pathology

**Correct answer:** C. RIM may extend beyond the radiation field.

## Which of the following diagnostic tests is recommended when evaluating for potential RIM in a woman with a history of breast cancer?

A. Ultrasound of the breast
B. Punch biopsy of affected skin
C. Serum anti-nuclear antibody
D. Serum anti-scleroderma-70 (topoisomerase 1) antibody
E. No diagnostic tests indicated

**Correct answer:** B. Punch biopsy of affected skin.
